# LC-MS/MS untargeted lipidomics uncovers placenta lipid signatures from intrahepatic cholestasis of pregnancy

**DOI:** 10.3389/fphys.2024.1276722

**Published:** 2024-06-03

**Authors:** Liling Xiong, Mi Tang, Hong Liu, Jianghui Cai, Ying Jin, Cheng Huang, Shasha Xing, Xiao Yang

**Affiliations:** ^1^ Obstetrics Department, Chengdu Women’s and Children’s Center Hospital, School of Medicine, University of Electronic Science and Technology of China, Chengdu, China; ^2^ GCP Institution, Chengdu Women’s and Children’s Center Hospital, School of Medicine, University of Electronic Science and Technology of China, Chengdu, China; ^3^ Department of Pharmacy, Chengdu Women’s and Children’s Center Hospital, School of Medicine, University of Electronic Science and Technology of China, Chengdu, China; ^4^ Clinical Lab, Chengdu Women’s and Children’s Center Hospital, School of Medicine, University of Electronic Science and Technology of China, Chengdu, China

**Keywords:** intrahepatic cholestasis of pregnancy, lipidomics, phosphatidylethanolamine, sphingolipids, autophagy

## Abstract

**Aims:** Intrahepatic cholestasis of pregnancy (ICP) stands as the predominant liver disorder affecting pregnant women, with a prevalence ranging from 0.2% to 15.6%. While ICP is known to heighten the chances of perinatal mortality and morbidity, its pathogenesis remains elusive, and therapeutic options are limited. The objective of this study was to explore the characteristic lipid signature in placentas collected from normal pregnancies and those with mild and severe intrahepatic cholestasis of pregnancy. This research aims to clarify the pathogenesis and identify lipid biomarker for ICP through LC-MS/MS based lipidomic analysis.

**Methods and materials:** Placenta samples were collected from 30 normal pregnancy women and 30 mild and severe ICP women respectively. Women with normal pregnancy and ICP were recruit from April 2021 to July 2022 in Chengdu, China. And LC-MS/MS based lipidomic analysis was used to explore the characteristic placental lipids in mild and severe ICP.

**Results:** Fourty-four lipids were differentially expressed both in mild and severe ICP placenta. The pathway analysis revealed these lipids are mainly enriched in glycerophospholipid metabolism and autophagy pathway. Weighted correlation network analysis (WGCNA) identified the correlation network module of lipids highly related to ICP. Using multiple logistic regression analysis, we identified three and four combined metabolites that had an area under receiver operating characteristic curves (AUC) ≥ 0.90.

**Conclusion:** Our results systematically revealed the lipid signature in mild and severe ICP placenta. The results may provide new insight into the treatment and early prediction of ICP.

## Graphical Abstract

**Figure:**
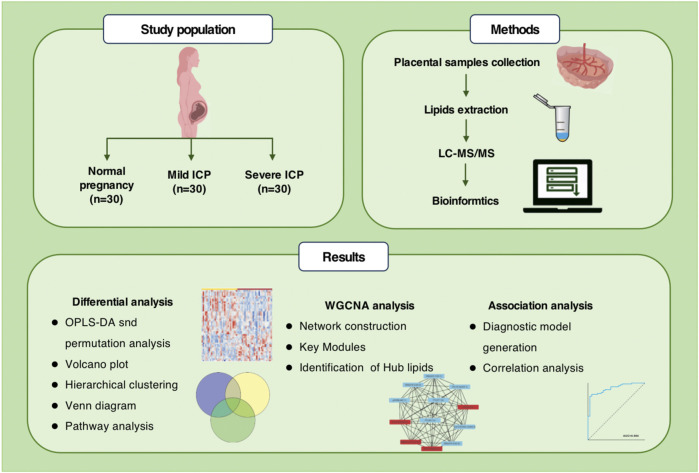
Overview of study design. Untargeted lipidomics workflow and data processing.

## 1 Introduction

Intrahepatic cholestasis of pregnancy (ICP) is the most common pregnancy-specific liver disease that usually occurs in the second or third trimester of pregnancy. Its clinical manifestations are mild to severe persistent pruritus, abnormal liver function, and elevated total bile acid (TBA) levels (Bicocca et al., 2018; Abdelhafez et al., 2022). While maternal risks are minimal with ICP, fetal complications such as premature delivery, meconium-stained amniotic fluid, neonatal depression, respiratory distress, and stillbirth can be significant (Glantz, Marschall and Mattsson, 2004). Global demographic data indicates ICP’s incidence varies from 0.1% to 15.6% (Gao et al., 2020). The therapeutic landscape for ICP remains challenging, though the utilization of ursodeoxycholic acid (UDCA) offers some relief. Clinical findings suggest UDCA alleviates pruritus and decrease serum bile acid concentrations but is still ambiguous concerning fetal protection (Chappell et al., 2019; Kumar and Kulkarni, 2020). Diagnostic criteria for ICP lack uniformity. Standard benchmarks incorporate pruritus combined with abnormal liver enzymes, spotlighting TBA levels as a diagnostic cornerstone. Proposals by the European Association for the Study of the Liver (EASL) and the Society for Maternal-Fetal Medicine (SMFM) pinpoint pruritus that abates post-delivery and TBA concentrations exceeding 10 μmol/L as diagnostic indicators (Lee, Mara, Metz and Pettker, 2021). Many prospective studies have revealed that TBA levels ≥40 μmol/L are associated with an increased risk of adverse neonatal outcomes in ICP (Geenes et al., 2014; Kawakita et al., 2015). Yet, relying solely on TBA metrics might be misleading. Retrospective study from Kondrackiene et al. emphasize the relevance of cholic acid (CA), chenodeoxycholic acid (CDCA), and the CA/CDCA ratio as superior diagnostic markers (Brites, Rodrigues, Oliveira, Cardoso and Graça, 1998; Kondrackiene and Kupcinskas, 2008). Hence, the pursuit for novel, efficacious diagnostic and prognostic markers in ICP is gaining traction.

The pathogenesis of ICP remains unknown, there are several theories about the causes of ICP, including the estrogen-bile acid axis, placental hypoxia, lipid metabolism disorders and trophoblast autophagy (Wei and Hu, 2014; Ozkan et al., 2015; Shan et al., 2021). As a subcategory of metabolic profiling, lipidomics can efficiently analyze lipid molecule changes in various pathophysiological processes (Han and Gross, 2022). More and more evidence proved that abnormal lipid metabolism is closely related to various pregnancy-related diseases such as preeclampsia, gestational diabetes mellitus, and preterm birth (Morillon et al., 2020; Wang et al., 2021; Zhang et al., 2022). Since ICP is closely related to bile acid metabolism, metabolomic technology has become an effective method for finding diagnostic markers in serum, urine, and even hair samples of ICP patients in recent years. Metabolomic signatures from serum and urine can be used as biomarkers for the diagnosis of ICP (Cui et al., 2018; Li et al., 2018). In recent years, more and more studies believe that lipid metabolism is closely related to the occurrence and development of ICP, the dysregulation of bile acid in serum and placenta will lead to abnormal lipid metabolism to a large extent, and then increase the risk of ICP (Hao et al., 2016). High triglyceride concentrations in the second and third trimesters have been reported to be associated with an increased risk of ICP, possibly due to reduced activity of bile acid receptors FXR and TGR5 (Fiorucci et al., 2009). A more recent untargeted lipidomics study revealed the abnormal lipid profiles of plasma collected from ICP patients and suggests that sphingolipid metabolism dysregulation may be associated with the development of ICP (Sun et al., 2022). Therefore, using lipid metabolites as clues may help us to further explore the molecular mechanisms underlying the occurrence of intrahepatic cholestasis of pregnancy.

This study aimed to compare the placenta lipid profiles in women with normal pregnancies and those with mild or severe ICP using LC-MS/MS based untargeted lipidomics. Through this approach, we pinpointed a distinct lipid signature offering a high diagnostic precision for ICP. This discovery may shed light on understanding ICP’s pathogenesis and enhancing its predictive accuracy.

## 2 Materials and methods

### 2.1 Clinical specimen collection and preparation

The placenta tissue was obtained from Chengdu Women’s and Children’s Central Hospital from April 2021 to July 2022 in Chengdu, China. The Ethics Committee of the Chengdu Women’s and Children’s Central Hospital approved the study documents and placenta collection (Permission Number: 2022 (49)-2).

The inclusion criteria of ICP were as follows: 1) new-onset pruritus and elevated levels of TBA, TBA level >10 but <40 μmol/L were categorized as mild, whereas the TBA level ≥40 μmol/L were categorized as severe; 2) age between 18 and 35 years; 3) singleton pregnancy; 4) providing informed consent. In this study, 30 severe ICP patients (ICP-S group), 30 mild ICP patients (ICP-M group) and 30 normal pregnant women (control group) who delivered via cesarean section were recruited from the Chengdu Women’s and Children’s Central Hospital. Patients who had received infertility treatment (such as *in vitro* fertilization or intrauterine insemination), serious illnesses before and during pregnancy (such as chronic liver and gall bladder diseases, skin disease, hypertension, preeclampsia, diabetes, hematological diseases, kidney, and nervous system disease), and infectious diseases (such as viral hepatitis, syphilis, or acquired immune deficiency syndrome) were excluded. The villous tissue of maternal placental samples (3 × 3 × 3 cm) was obtained immediately after delivery. Preservation of these samples involved instant freezing using liquid nitrogen, followed by storage at a temperature of −80°C, ensuring their readiness for lipidomics evaluations.

### 2.2 Sample preparation

Lipid extraction was conducted using a meticulously designed protocol. Initially, placental samples weighing 30 mg were treated with a mixture of methanol and water. To ensure extraction consistency, 20 μL of LysoPC-17:0 at 0.1 mg/mL concentration (previously dissolved in methanol) was incorporated as an internal benchmark. Two steel balls were then added to the mixture and underwent grinding. In the subsequent step, each sample received 300 μL of chloroform, followed by ultrasonic extraction. The samples were later stored at −20°C for a span of 20 min. Before moving to the next phase, centrifugation was done at a speed of 13,000 rpm at 4°C, lasting 10 min. Post-centrifugation, 200 μL of the supernatant was carefully decanted into specific sample containers. The residue then saw an addition of 300 μL chloroform combined with methanol in a 2:1 volume ratio, enhanced with 0.1 mM BHT. After vortexing these samples for 30 s, a 10-min ultrasonic extraction was performed in an ice-water bath. The mixture was then chilled at −20°C for 20 min. Centrifugation was repeated under previously mentioned conditions. The resultant supernatants from the two phases were amalgamated and stirred thoroughly. The combined liquid (400 μL) underwent evaporation under a nitrogen stream and was reconstituted in a mixture of isopropanol and methanol. This was vortexed for 30 s and subjected to a 3-min ultrasonic extraction in an ice-water environment. Prior to further assessments, it was passed through a 0.22 μm filter specifically designed for organic phases. Quality control (QC) specimens were formulated by taking portions of every sample and creating a unified pooled sample.

### 2.3 Untargeted lipidomics analysis

Untargeted lipidomics as an important branch of untargeted metabolomics, is a new subject for the systematic analysis of lipids *in vivo*. Based on LC-MS, all lipid molecules in biological samples such as cells, tissues, organs or body fluids were detected as much as possible without bias. Comparative analysis is conducted between the experimental group and the control group, and differential lipid branches are screened through statistical analysis to find the relative relationship between lipid metabolism changes and physiological and pathological changes, revealing the mechanism of lipid metabolism in various life activities. LC-MS/MS investigations were conducted on a Dionex Ultimate 3000 RS UHPLC system, which was connected to a Q-Exactive quadrupole-Orbitrap mass spectrometer, outfitted with a heated ESI source (provided by Thermo Fisher Scientific, Waltham, MA, USA). Chromatographic separations were achieved using an ACQUITY UPLC BEH C18 column (1.7 μm, 2.1 × 100 mm), operating in both anion and cation detection modes. We employed a binary solvent gradient system, where solvent (A) was an acetonitrile:water blend (60:40, v:v) supplemented with 10 mmol/L ammonium formate, and solvent (B) was a mixture of acetonitrile and isopropanol in the ratio 10:90, v:v, also containing 10 mmol/L ammonium formate. The gradient schedule was as follows: from 0 to 0.5 min, 5% B; at 2 min, 43% B; 32.1 min, 52% B; 8.5 min, 53% B; 8.6 min, 75% B; 114 min, 90% B; 14.5 min, it was 100% B; 15.5 min, maintaining at 100% B; 15.7 min, reverting to 5% B; and at 18 min, remaining at 5%B. The chromatographic conditions included a flow rate of 0.4 mL/min and a column oven temperature of 60°C. Throughout the analytical procedure, samples were stabilized at 4°C. The injector was set to deliver 5 μL of each sample. Operating the mass spectrometer, both positive ESI+ and negative ESI- ionization modes were utilized. To ensure consistency and reliability of the results, QC samples were introduced at consistent intervals during the analysis.

### 2.4 Data preprocessing and statistical analysis

Raw data from the Q Exactive LC-MS/MS were processed using Lipid Search software to analyze MSn and determine the precise m/z ratio of precursor ions. The molecular configuration of lipids, along with the ionization modes of both positive and negative ions, was discerned based on the precursor ions and the multistage mass spectrometry results from each sample. These results were then aligned within a specific retention time window and consolidated to produce a singular data matrix report. Within every sample, the signal intensities of all peaks underwent normalization. Specifically, the intensity of every peak was transformed into its relative spectral intensity, and this value was multiplied by 10,000. The refined data had peaks with missing values in over half the groups (ion intensity equals zero) removed and zero values substituted by half of the lowest recorded value. Data from both ion modes were then integrated into a singular matrix. This matrix was introduced into the R environment for PCA, offering insights into sample distribution and ensuring analytical process stability. Both OPLS-DA and PLS-DA methods were applied to highlight the distinct metabolites between groups. To counteract potential model overfitting, the model’s quality was verified using 7-fold cross-validation alongside 200 iterations of RPT.

From the OPLS-DA model, VIP (Variable Importance of Projection) values facilitated the prioritization of each variable’s role in distinguishing between groups. To delve into metabolic pathway enrichment, we referred to the KEGG (Kyoto Encyclopedia of Genes and Genomes) database. Using the R package as described by Langfelder and Horvath (2008), we structured a weighted metabolite co-expression network. Here, a co-expression module denotes a cluster of metabolites showcasing a pronounced topological overlap similarity. Correlations between these modules and clinical characteristics were ascertained using Pearson correlation analysis. Modules registering a *p*-value under 0.05 held statistically significant differences. In our pursuit of a diagnostic model, we harnessed the capabilities of ROC (receiver operating characteristic) curves, and utilized multiple logistic regression to amalgamate several markers. Graphical representations were crafted with GraphPad Prism 9, data are presented as the mean ± SEM. Statistical data were analyzed by Student’s t-test (2 groups) and one-way ANOVA (>2 groups). Notably, lipids that carried a VIP value exceeding 1.0 and a *p*-value below 0.05 were flagged as undergoing significant alterations.

## 3 Results

### 3.1 Clinical characteristics of women with ICP and normal pregnant women

Table 1 outlines the clinical parameters of both ICP patients and women with normal pregnancies. No noticeable difference emerged in factors such as maternal age, BMI, gravidity, parity, and placental mass between the two groups (*p* > 0.05). Yet, it was observed that the gestational age at the time of delivery appeared shorter in women diagnosed with either mild or severe ICP. Notably, parameters like TBA, ALT, and AST levels exhibited elevated values in both mild and severe ICP patients compared to those with normal pregnancies. Furthermore, the birth weight of neonates born to ICP mothers was discernibly lower than that of their counterparts born to healthy mothers, and the percentile of fetal birth weight was presented as a table in our (Supplementary Table S1).

**TABLE 1:** Clinical characteristics of the participants.

Variables	Normal pregnancy	Mild ICP	*P*	Severe ICP	*P*
Sample size (n)	30	30		30	
Maternal age (years)	31.00 ± 0.61	29.40 ± 0.64	0.076	29.43 ± 0.66	0.087
BMI (kg/m^2^)	26.47 ± 0.46	25.30 ± 0.47	0.080	25.61 ± 0.46	0.193
Gravity no. (%)			0.107		0.057
1	7 (23.3)	14 (46)		16 (53)	
2	15 (50)	8 (27)		9 (30)	
≥3	8 (26.7)	8 (27)		5 (17)	
Parity no. (%)			0.304		0.138
0	15 (50)	20 (67)		22 (73)	
1	14 (47)	10 (33)		8 (27)	
≥2	1 (3)	0 (0)		0 (0)	
Gestational age at delivery (weeks)	38.80 ± 0.10	37.93 ± 0.22^**^	<0.01	37.57 ± 0.23^**^	<0.01
Birth weight (g)	3,434 ± 77.69	3,123 ± 66.29^**^	<0.01	2,996 ± 82.69^**^	<0.01
Placenta weight (g)	512.7 ± 7.00	505.0 ± 11.27	0.566	496.3 ± 11.81	0.239
TBA (μmol/L)	3.1 (2.1–4.5)	15.9 (11.7–21.3)^**^	<0.01	56.0 (43.0–75.7)^**^	<0.01
ALT (IU/L)	16.6 (12.5–22.2)	93.4 (38.5–123.8)^**^	<0.01	180.2 (103.0–343.8)^**^	<0.01
AST (IU/L)	20.0 (17.0–23.3)	46.9 (31.0–69.7)^**^	<0.01	140.3 (66.9–274.2)^**^	<0.01

Data are presented as mean ± SEM, median (interquartile range), or n (%). Abbreviations: ICP, intrahepatic cholestasis of pregnancy; BMI, body mass index; TBA, total bile acid; TBIL, total bilirubin; DBIL, direct bilirubin; ALT, alanine aminotransferase; AST, aspartate aminotransferase; ALP, alkaline phosphatase. *p < 0.05, **p < 0.01. The *p* values in the table were mild ICP, and severe ICP, compared with the control group respectively.

### 3.2 The lipid profiles of placenta collected from ICP patients

**FIGURE 1:**
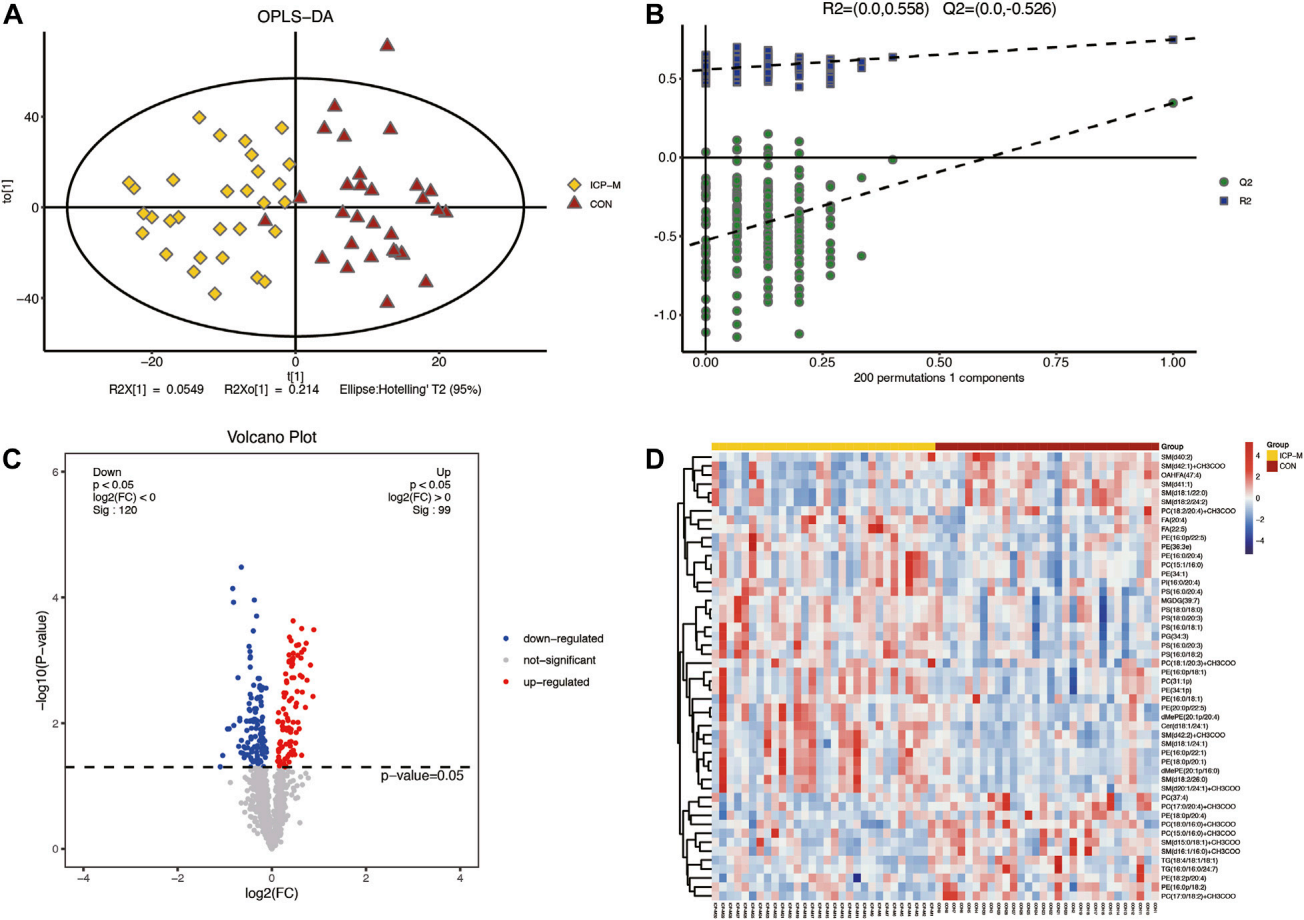
Comparative LC-MS/MS lipidomic insights between mild ICP cases and the control cohort. **(A)** Supervised OPLS-DA score plot contrasts mild ICP (highlighted in yellow) against the control samples (depicted in red). **(B)** Permutation assessment. R2x (cum) and R2y (cum) elucidate the cumulative explanatory power along the x and y dimensions. “Cum” abbreviates the aggregated contribution from multiple principal components. The Q2 (cum) value signifies the model’s cumulative predictive capability. **(C)** Differential lipid expression visualized through volcano plots. Elevated levels are colored in red, diminished levels in blue, and those without statistical variance are marked in gray. **(D)** Hierarchical sample clustering provides insights into lipid expression variances.

**FIGURE 2:**
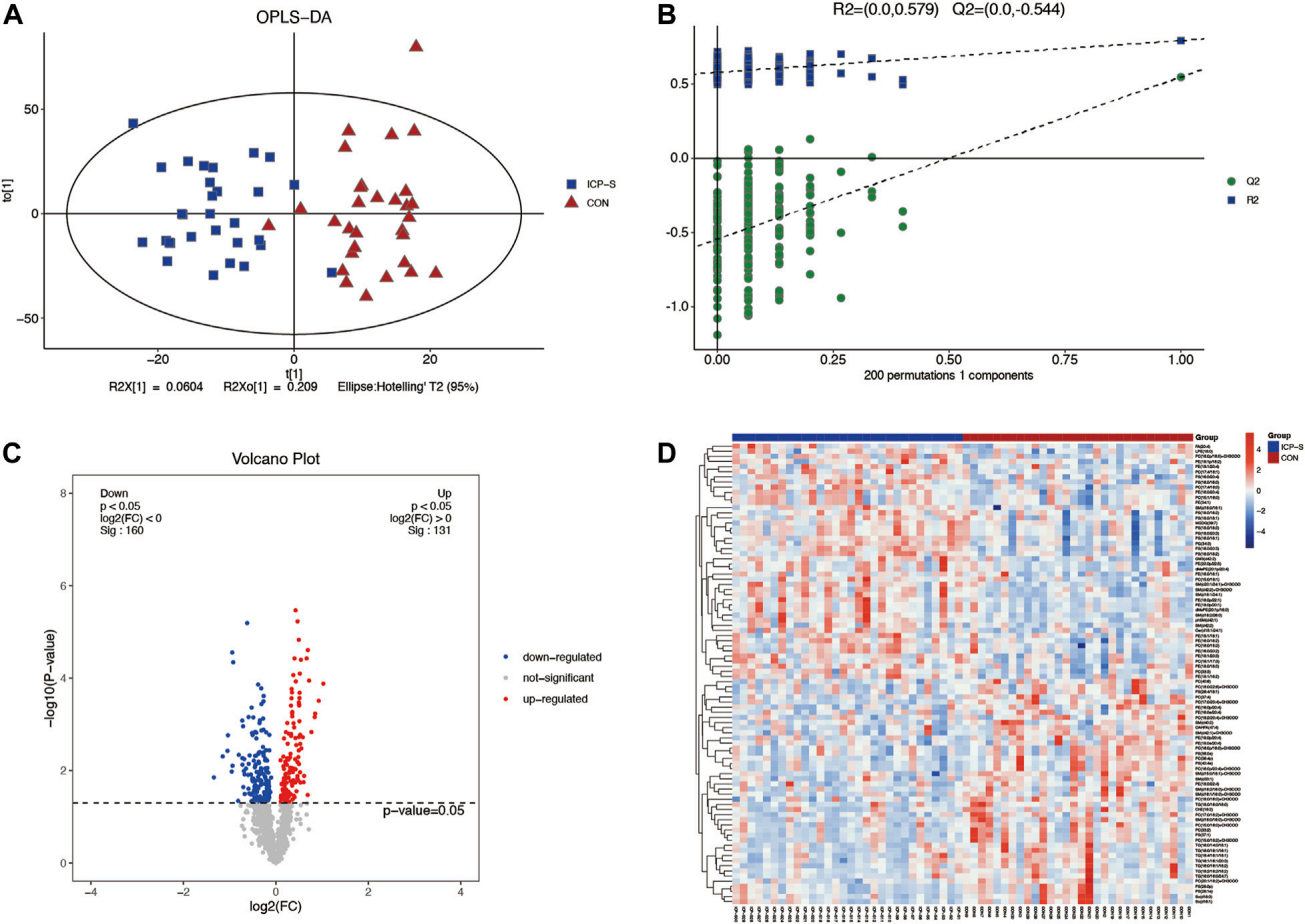
Differential LC-MS/MS lipidomic profile contrasting severe ICP cases with the control set. **(A)** OPLS-DA score chart comparing severe ICP (denoted in blue) against controls (marked in red). **(B)** Evaluation via permutation. Both R2x (cum) and R2y (cum) define the model’s cumulative interpretative capacity along their respective x and y coordinates. The term ‘Cum’ alludes to the aggregated input from distinct principal components. Predictive accuracy of the model is indicated by Q2 (cum). **(C)** A depiction of lipid variations using volcano plots. Elevations are marked red, depressions in blue, and lipids without noteworthy statistical disparity are shaded gray. **(D)** Cluster analysis visually representing lipidomic variations across individual sample sets.

Lipidomic analyses were performed on placenta samples utilizing UHPLC-Q Exactive MS as detailed in our methods. Lipid profiles were individually assessed across 30 cases each of mild and severe ICP, contrasted against 30 cases of normal pregnancies. Using OPLS-DA, we undertook multivariate statistical approaches to discern overarching discrepancies in lipid metabolites across the groups. Clear separations between normal cases and both mild and severe ICP cases were evident in the OPLS-DA score scatter plot (Refer to Figure 1A and Figure 2A). Specifically, samples of both ICP severities diverged from controls primarily along the x-coordinate. The model’s precision was authenticated via permutation testing. Notably, all left-positioned Q2 values (in green) were diminished compared to their right-sided counterparts. Further, the Q2 regression line’s intersection with the vertical on the left falls below zero, underscoring the model’s potency and predictive reliability (See Figure 1B and Figure 2B).

A total of 61 lipids (VIP >1 and *p* < 0.05) were found to be significantly different between mild ICP and control groups, which included 27 downregulated and 34 upregulated lipids in the ICP-M group. The expression levels of the 61 differential lipids were used to draw a volcano plot (Figure 1C), the blue dots represent remarkably downregulated lipids, while the red dots represent significantly upregulated lipids. The relative contents of the top 50 differentially expressed lipids were observed through a hierarchical cluster heatmap (Figure 1D). All of the differentially expressed lipids can be classified into 16 categories, including: 15 PE species, 10 PC species, seven PS species, 12 SM species, 3 TG species, two FA species, two dMePE species, and one OAHFA, MGDG, Cer, GM3, phSM, LPE, ChE, PG, PI species. Detailed information about these differential lipids is shown in Supplementary Table S2.

We also obtained 88 differential lipids between severe ICP and the control group according to the conditions of VIP >1 and *p* < 0.05. A total of 46 lipids exhibited increased levels, and 42 exhibited decreased levels in the ICP-S group, and the detailed information between these two groups was shown in Supplementary Table S3. The heatmap and volcano plots were conducted to display differences in lipid metabolites between the two groups (Figures 2C, D). All the 88 differential lipids can be classified into 17 categories, including 20 PC species, 19 PE species, 15 PS species, 13 SM species, 7 TG species, two dMePE species, two So species, and one OAHFA, MGDG, Cer, GM3, phSM, LPE, ChE, PG, PI, FA species, respectively.

### 3.3 Critical lipids screening through venn diagram and pathway analysis

**FIGURE 3:**
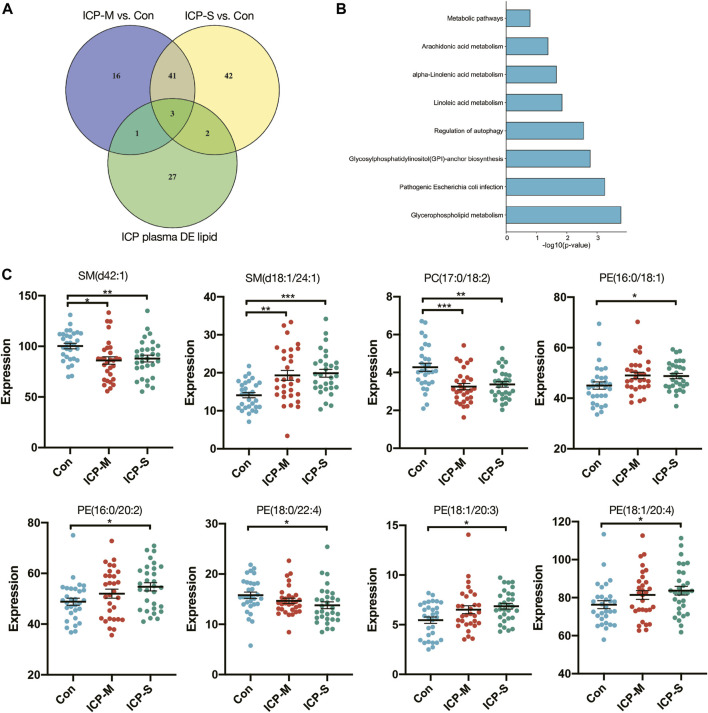
Differentially expressed lipids in the mild and severe group and the pathway analysis. **(A)** Venn diagram of the differentially expressed lipids in ICP placenta and ICP plasma. **(B)** Pathway analysis of 44 differentially altered lipids. **(C)** Intensity of differential lipids expression in the control (blue), mild ICP (red) and severe ICP (green). Data are presented as the means ± SEMs. Data were analyzed using one-way ANOVA. *p < 0.05, **p < 0.01, ***p < 0.0001 vs. the control group.

We identified 44 lipids differentially expressed both in the mild and severe ICP group compared with the control group (Figure 3A). Detailed information regarding the 44 differential lipids was shown in Table 2. To screen critical lipids that are associated with ICP, we combined the previously published ICP plasma lipidomics data for further analysis (Sun et al., 2022). In the previously reported data, Sun et al. revealed differentially expressed plasma lipids in ICP patients, and we identified three differential lipids co-expressed both in ICP placenta and ICP plasma (Figure 3A). These three lipids are SM (d42:1), SM (d18:1/24:1) and PC (17:0/18:2), and their expression level in the ICP placenta is significantly altered (Figure 3C). Based on the identified 44 differential lipids, we conducted a pathway analysis to reveal possible metabolic pathways involved in ICP onset. The results of the pathway analysis show that the significantly altered lipids were enriched in autophagy regulation, glycerophospholipid metabolism and GPI-anchor biosynthesis (Figure 3B). It was reported autophagy is closely related to ICP pathogenesis (Hu et al., 2018; Shan et al., 2021; Dong et al., 2023), we further analyzed the individual autophagy pathways and found 5 PE lipids are closely in correlation with autophagy (Supplementary Figure S1), including PE (16:0/18:1), PE (16:0/20:2), PE (18:1/20:3), PE (18:1/20:4), exhibited increased level in ICP placenta, while PE (18:0/22:4) exhibited decreased expression level (Figure 3C), indicate these lipids may play a pivotal role in the occurrence and development of ICP.

**TABLE 2:** List of the 44 differential lipids in both mild and severe ICP placenta.

Class	Lipid	Formula
Sphingomyelin (SM)	SM(d42:1)	C_47_H_95_O_6_N_2_P_1_
	SM(d18:1/24:1)	C_47_H_93_O_6_N_2_P_1_Na_0_
	SM(d15:0/18:1)	C_38_H_77_O_6_N_2_P_1_
	SM(d18:2/26:0)	C_49_H_97_O_6_N_2_P_1_
	SM(d40:2)	C_45_H_89_O_6_N_2_P_1_
	SM(d20:1/24:1)	C_49_H_97_O_6_N_2_P_1_
	SM(d16:1/16:0)	C_37_H_75_O_6_N_2_P_1_
	SM(d33:1)	C_38_H_77_O_6_N_2_P_1_
Phosphatidylethanolamine (PE)	PE (18:0p/20:4)	C_43_H_78_O_7_N_1_P_1_
	PE (16:0/20:4)	C_41_H_74_O_8_N_1_P_1_
	PE (34:1)	C_39_H_76_O_8_N_1_P_1_Na_0_
	PE (16:0/18:1)	C_39_H_76_O_8_N_1_P_1_
	PE (18:0p/20:1)	C_43_H_84_O_7_N_1_P_1_
	PE (20:0p/22:5)	C_47_H_84_O_7_N_1_P_1_
	PE (16:0p/22:1)	C_43_H_84_O_7_N_1_P_1_
	PE (18:1/20:3)	C_43_H_78_O_8_N_1_P_1_
Phosphatidylcholine (PC)	PC(15:1/16:0)	C_39_H_76_O_8_N_1_P_1_Na_0_
	PC(17:0/18:2)	C_43_H_82_O_8_N_1_P_1_
	PC(15:0/16:0)	C_39_H_78_O_8_N_1_P_1_
	PC(37:4)	C_45_H_82_O_8_N_1_P_1_
	PC(17:0/20:4)	C_45_H_82_O_8_N_1_P_1_
	PC(33:2)	C_41_H_78_O_8_N_1_P_1_
	PC(15:0/18:2)	C_41_H_78_O_8_N_1_P_1_
Phosphatidylserine (PS)	PS(16:0/18:1)	C_40_H_76_O_10_N_1_P_1_
	PS(16:0/20:4)	C_42_H_74_O_10_N_1_P_1_
	PS(18:0/18:0)	C_42_H_82_O_10_N_1_P_1_Na_0_
	PS(18:0/20:3)	C_44_H_80_O_10_N_1_P_1_
	PS(37:1)	C_43_H_82_O_10_N_1_P_1_
	PS(16:0/20:3)	C_42_H_76_O_10_N_1_P_1_
	PS(16:0/18:2)	C_40_H_74_O_10_N_1_P_1_
Triradyglycerols (TG)	TG (18:4/18:1/18:1)	C_57_H_98_O_6_
	TG (16:0/16:0/24:7)	C_59_H_100_O_6_
	TG (18:0/16:0/16:0)	C_53_H_102_O_6_N_0_
Phosphatidylglycerol (PG)	PG (34:3)	C_40_H_73_O_10_N_0_P_1_
Lyso-phosphatidylethanolamine (LPE)	LPE (18:0)	C_23_H_48_O_7_N_1_P_1_
Dimethylphosphatidylethanolamine (dMePE)	dMePE (20:1p/16:0)	C_43_H_84_O_7_N_1_P_1_
	dMePE (20:1p/20:4)	C_47_H_84_O_7_N_1_P_1_
Fatty acid (FA)	FA (20:4)	O_2_H_32_C_20_
(O-acyl)-1-hydroxy fatty acid (OAHFA)	OAHFA (47:4)	C_47_H_84_O_4_
Monogalactosyldiacylglycerol (MGDG)	MGDG (39:7)	C_48_H_78_O_10_
Ceramides (Cer)	Cer(d18:1/24:1)	C_42_H_81_O_3_N_1_
Ganglioside (GM3)	GM3(d42:2)	C_65_H_118_O_21_N_2_
Sphingomyelin (phytosphingosine) (phSM)	phSM(d42:1)	C_47_H_95_O_7_N_2_P_1_
Cholesteryl Ester (ChE)	ChE(18:2)	C_45_H_76_O_2_N_0_

### 3.4 Critical lipid Co-expression network modules closely correlated with ICP

**FIGURE 4:**
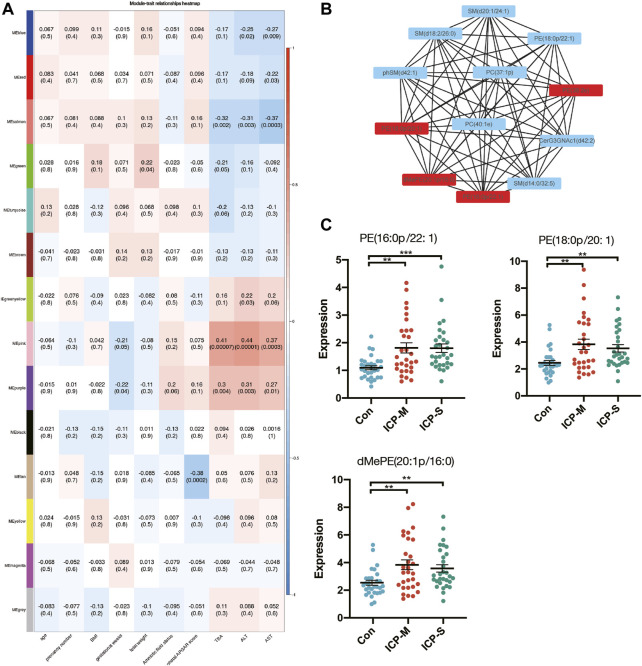
WGCNA analysis and identification of lipids associated with the clinical traits of ICP. **(A)** Pearson correlation analysis of the lipids module and clinical traits, the red color indicates a positive correlation and blue indicates negative correlation. The depth of the color indicates the strength of the correlation. **(B)** Hub lipids in pink module was screened out by CytoScape. **(C)** Intensity of differential hub lipids expression in the pink module. Data are presented as the means ± SEMs. Data were analyzed using one-way ANOVA. **p < 0.01, ***p < 0.0001 vs. the control group.

To elucidate the ties between lipid profiles and clinical traits within our study cohort, we embarked on a Weighted Gene Co-expression Network Analysis (WGCNA). This aimed to pinpoint clusters or modules marked by analogous expression trajectories. In this analysis, we obtained 14 modules and the result showed that most lipids were clustered in turquoise, yellow and pink modules (Supplementary Figure S2). Among the 14 modules, the pink module and purple module showed a significant positive correlation with TBA, ALT and AST levels, whereas the salmon module negatively correlated with TBA, ALT and AST levels (Figure 4A). A total of 30 lipids were included in these three modules, and we identified 11 differentially co-expressed lipids overlapped with the dysregulated lipids in both mild and severe ICP group (Supplementary Figure S3A). Next, the CytoScape was used to screen hub lipids in pink, purple and salmon module based on the MCC algorithm. The top 4 lipids in the MCC algorithm were identified as hub lipids. As shown in Figure 4B, we identified PE (18:0p/20:1), dMePE (20:1p/16:0), PE (16:0p/22:1) and PE (38:2e) as hub lipids in the pink module. Among these four hub lipids, the expression level of PE (18:0p/20:1), dMePE (20:1p/16:0) and PE (16:0p/22:1) was significantly elevated in the mild and severe ICP group (Figure 4C). The network diagram also depicts the hub lipids in purple and salmon module (Supplementary Figures S3B, C).

### 3.5 Diagnostic utility of ICP placenta differential lipids

**FIGURE 5:**
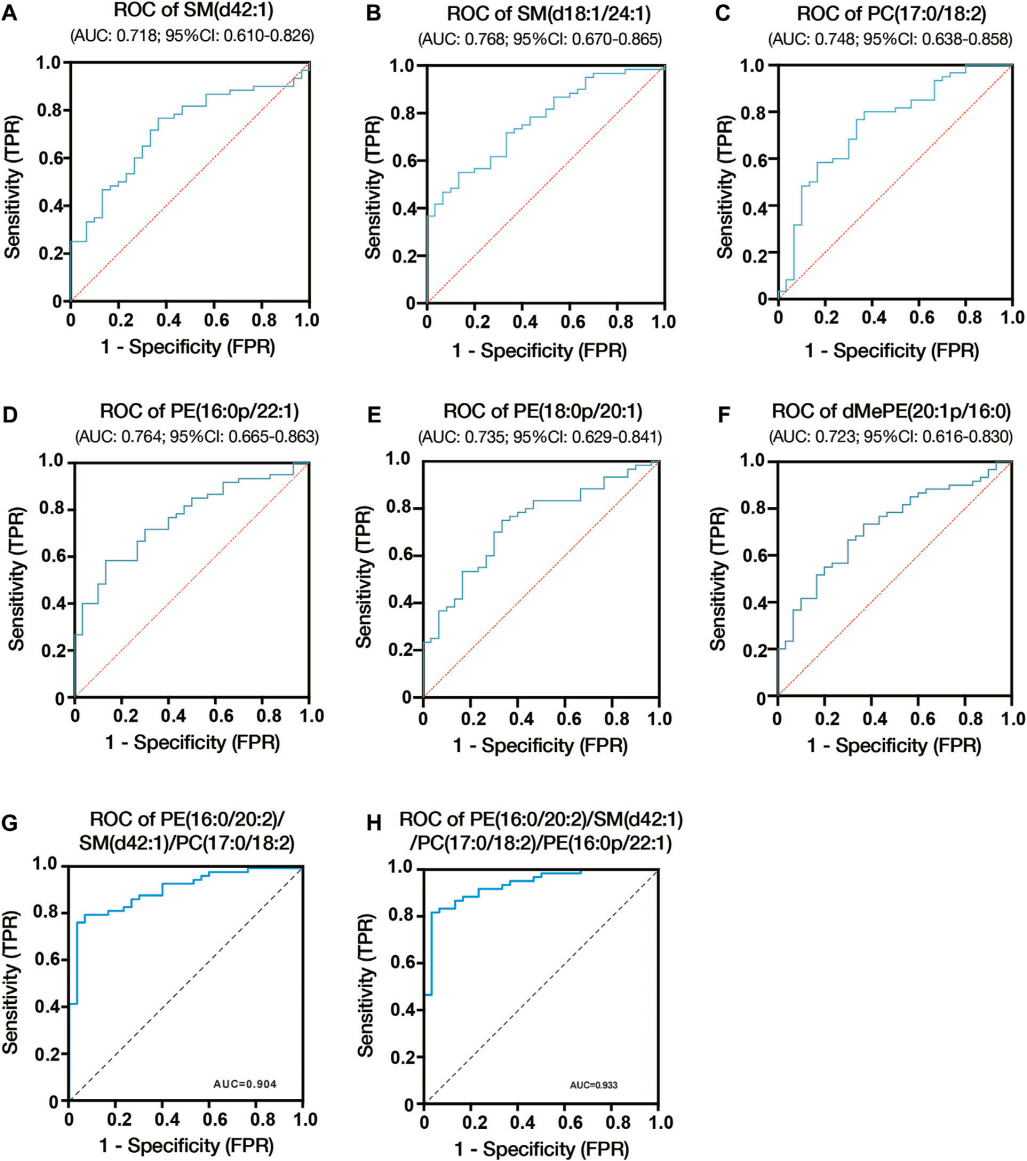
Diagnostic utility of differential lipids in placenta from ICP pregnant women. **(A)** ROC analysis of SM (d42:1). **(B)** ROC analysis of SM (d18:1/24:1). **(C)** ROC analysis of PC (17:0/18:2). **(D)** ROC analysis of PE (16:0p/22:1). **(E)** ROC analysis of PE (18:0p/20:1). **(F)** ROC analysis of dMePE (20:1p/16:0). Multiple logistic regression analysis showed the combined ROC analysis of PE (16:0/20:2)/SM (d42:1)/PC (17:0/18:2) **(G)** and PE (16:0/20:2)/SM (d42:1)/PC (17:0/18:2)/PE (16:0p/22:1) **(H)**. AUC, area under the curve; CI, confidence interval; ROC, receiver operating characteristic.

We constructed ROC curves and calculated AUCs to evaluate the probability of these lipids being diagnosed with ICP. As shown in Figures 5A–C, the AUCs of SM (d42:1), SM (d18:1/24:1) and PC (17:0/18:2) were 0.718, 0.768 and 0.748, respectively. And the AUCs of PE (18:0p/20:1), dMePE (20:1p/16:0) and PE (16:0p/22:1) were 0.735, 0.723 and 0.764 (Figures 5D–F). We also evaluate the diagnostic utility of 5 lipids identified from autophagy pathway analysis, results showed the AUCs of PE (16:0/18:1), PE (16:0/20:2), PE (18:0/22:4), PE (18:1/20:3) and PE (18:1/20:4) were 0.682, 0.649, 0.665, 0.659 and 0.657, respectively (Supplementary Figure S4). Through comprehensive multiple logistic regression analysis, it emerged that an efficacious diagnostic model for ICP could emerge by integrating these lipid profiles. Analysis results of combined biomarker data yielded an AUC of 0.904 for PE (16:0/20:2), SM (d42:1) and PC (17:0/18:2) (Figure 5G). The AUCs even reach 0.933 when combining PE (16:0/20:2), SM (d42:1), PC (17:0/18:2) and PE (16:0p/22:1) (Figure 5H). Therefore, the combination of these lipids could be reliable, and a novel biomarker in predicting the risk of ICP.

## 4 Discussion

The etiology of Intrahepatic cholestasis of pregnancy (ICP) remains elusive, despite its association with heightened perinatal mortality and morbidity. Presently, the therapeutic landscape for ICP lacks efficient interventions; once confirmed, prompt delivery becomes the sole viable preventive measure against adverse outcomes in pregnancy (Mathur et al., 2022). Branching out from metabolomics, lipidomics emerges as a budding domain, increasingly illuminating the multifunctional nature of lipids in a plethora of biological systems. Prior research alludes to variances in placental lipid constituents, such as LDL cholesterol and sphingolipids, when contrasting normal pregnancies with those complicated by ICP (Dann et al., 2006; Sun et al., 2022). Acknowledging the conceivable role of lipid metabolic irregularities during gestation, delving deeper into lipid metabolic pathways might pave the way for enhanced diagnostic and therapeutic avenues for ICP.

In this study, we found ICP placenta has a significantly different lipid profile from normal pregnancy. We found the differentially expressed lipids in mild ICP placenta mainly include PE, PC, PS and SM species. We also identified differential lipids between severe ICP placenta and normal pregnancy, and these lipids mainly include PE, PC, PS, SM and TG species. Among these lipids, we identified 44 lipids differentially expressed both in mild and severe ICP group compared with the control group, mainly of them were SM, PE, PC and PS species. Moreover, pathway analysis of the placentas of patients with ICP identified autophagy regulation, glycerophospholipid metabolism and GPI-anchor biosynthesis, suggesting these pathways might be related to the pathogenesis of ICP.

Autophagy, an ancient cellular mechanism conserved through evolution, targets a myriad of cytoplasmic components like organelles and protein aggregates for degradation within lysosomes, ensuring cellular homeostasis during external stress conditions (Cao et al., 2021; Klionsky et al., 2021). Autophagy plays a pivotal role in implantation, embryogenesis and maintenance of pregnancy (Nakashima et al., 2019). A previous study demonstrated the expression of LC3-II is elevated in ICP human placenta and placentas of ICP rats, indicating autophagy is closely related to ICP (Hu et al., 2018). Zhang et al. used TCA-treated term placental villous for proteomic analysis, their results revealed protein expression difference in ICP mainly related to autophagy and cell metabolism (Zhang et al., 2013). Consistent with their result, Fang et al. conducted quantitative proteomics and their results showed the differentially expressed proteins in ICP placenta mainly participated in autophagy, autophagosome formation and metabolism (Fang et al., 2022). All of these indicate a close correlation between ICP and autophagy. In our lipidomic analysis, we also found the differentially expressed lipids mainly enriched in the autophagy pathway, and PE (16:0/18:1), PE (16:0/20:2), PE (18:1/20:3), PE (18:1/20:4) and PE (18:0/22:4) participated in this process. Phosphatidylethanolamine (PE) is the second most abundant phospholipid in the membranes of all mammalian cells (Vance, 2008; van der Veen et al., 2017). As a fundamental component of biological membranes, PE is essential for many cellular functions and the dysregulation of PE metabolism has been implicated in many diseases, such as nonalcoholic liver disease, atherosclerosis and obesity (Fu et al., 2011; Calzada et al., 2016). In addition, PE is essential for the formation of LC3-II in the autophagolysosomal bilayer membrane. During autophagic events, cytosolic LC3-I associates with phosphatidylethanolamine, yielding LC3-II, which integrates into autophagosomal membranes. The prevalence of this marker, LC3-II, offers insight into autophagic vigor, making it a dependable autophagy monitor (Runwal et al., 2019). Consequently, an overabundance of PE lipids like PE (16:0/18:1), PE (16:0/20:2), PE (18:1/20:3) and PE (18:1/20:4) in ICP placentas might stimulate the progression of LC3-II and prompt the autophagy circuit, which empirical studies affirm as contributory to ICP’s genesis and progression.

Through combined analysis with previous studies, we also identified SM (d42:1), SM (d18:1/24:1) and PC (17:0/18:2) are differentially expressed both in ICP placenta and plasma samples. And their good diagnostic utility was also accessed through the construction of ROC curves. Two of these three lipids were sphingolipids. The sphingolipid metabolic pathway produces bioactive metabolites like sphingosine 1-phosphate (S1P), ceramide and sphingosine, they can participate in various biological processes such as cell survival, growth, vascular integrity and inflammation. Sphingolipid disruptions have been tied to adverse pregnancy outcomes like preeclampsia, recurrent pregnancy loss and intrauterine growth restriction (Chauvin et al., 2015; Del Gaudio et al., 2020; Wang et al., 2021). A previous study reported the median values of C16-Cer and C18-Cer were significantly increased in the plasma of ICP patients, and after treatment with UDCA for a week, the concentration of C16-Cer and C18-Cer decreased considerably (Mikucka-Niczyporuk et al., 2020). Consistent with their finding, Sun et al. also reported ICP is associated with disordered sphingolipid homeostasis (Sun et al., 2022). Collectively, this evidence proved that sphingolipids may act as new diagnostic targets for ICP.

There are still several limitations in our study. First, further animal and cellular experiments are needed to elucidate the specific mechanisms of lipid changes. Second, many studies on lipidomics and ICP have been conducted with a small number of patients, which limits the generalizability of the findings. Larger studies are needed to confirm the role of lipids in ICP. Thirdly, the percentile of the fetal birth weight varied among groups, there are some values outside the 10th to 90th percentiles. Although small or large fetal size does not necessarily indicate pathological status, fetal growth restriction (FGR) and fetal overgrowth may result from abnormal placental supplies. There is a lack of parameters for placental function (e.g., Doppler velocimetry measurements, growth trajectory and serum biomarkers) to distinguish FGR from SGA in our research, so the inclusion and exclusion criteria should be considered more comprehensive in the future. Finally, the diagnosis of ICP is currently based on clinical symptoms and biochemical tests. However, there is a lack of standardized criteria for the diagnosis of ICP, which can lead to variability in patient selection and make it challenging to compare results across studies.

In conclusion, we used UHPLC to characterize the placental lipidomics profiling for women complicated with ICP. Our results confirmed there were significant changes in lipid profile in the mid and severe ICP placenta, the metabolic pathway was primarily associated with autophagy and glycerophospholipid metabolism. And ICP placenta is associated with dysregulation of sphingolipid homeostasis. Our research highlighted some important mechanisms involved in the onset of ICP and might provide new insight into the treatment and diagnosis of ICP.

## Figures and Tables

**Table:** 

**ICP**	Intrahepatic cholestasis of pregnancy
**TBAs**	total bile acids
**UDCA**	ursodeoxycholic acid
**EASL**	The European Association for the Study of the Liver
**SMFM**	the Society for Maternal-Fetal Medicine
**CA**	cholic acid
**CDCA**	chenodeoxycholic acid
**ALT**	alanine aminotransferase
**AST**	aspartate aminotransferase
**LDL**	Low-density lipoprotein
**TC**	Total cholesterol
**TG**	Triglyceride
**UHPLC-Q**	Exactive MS Ultrahigh-performance liquid chromatography plus Q-Exactive mass spectrometry PC Phosphocholine
**LPC**	Lysophosphocholine
**IDL**	Intermediate-density lipoprotein
**LC-MS**	Liquid chromatography coupled with
**MS SM**	Sphingomyelin
**CE**	Electrophoresis
**PE**	Phosphatidylethanolamine
**PS**	Phosphatidylserine Cer Ceramide
**LPE**	Lysophosphatidylethanolamine
**CE**	Cholesteryl esters
**DAG**	Diacylglycerol
**ESI**	Electrospray ionization
**PA**	Phosphatidic acid
**PCA**	Principal component analysis
**OPLS-DA**	Orthogonal partial least squares-discriminant analysis
**PLS-DA**	Partial least squares-discriminant analysis
**VIP**	Variable importance of projection
**KEGG**	Kyoto Encyclopedia of Genes and Genomes

## Data Availability

The data presented in this study have been deposited in the Metabolights repository, with the identifier MTBLS9458. The complete dataset can be accessed here: www.ebi.ac.uk/metabolights/MTBLS9458.
