# Corrigendum: LC-MS/MS untargeted lipidomics uncovers placenta lipid signatures from intrahepatic cholestasis of pregnancy

**DOI:** 10.3389/fphys.2024.1454937

**Published:** 2024-07-24

**Authors:** Liling Xiong, Mi Tang, Hong Liu, Jianghui Cai, Ying Jin, Cheng Huang, Shasha Xing, Xiao Yang

**Affiliations:** ^1^ Obstetrics Department, Chengdu Women’s and Children’s Center Hospital, School of Medicine, University of Electronic Science and Technology of China, Chengdu, China; ^2^ GCP Institution, Chengdu Women’s and Children’s Center Hospital, School of Medicine, University of Electronic Science and Technology of China, Chengdu, China; ^3^ Department of Pharmacy, Chengdu Women’s and Children’s Center Hospital, School of Medicine, University of Electronic Science and Technology of China, Chengdu, China; ^4^ Clinical Lab, Chengdu Women’s and Children’s Center Hospital, School of Medicine, University of Electronic Science and Technology of China, Chengdu, China

**Keywords:** intrahepatic cholestasis of pregnancy, lipidomics, phosphatidylethanolamine, sphingolipids, autophagy

In the published article, there was an error in the **Funding** statement. The Grant/Award Number associated with Sichuan maternal and child medical science and Technology innovation project was incorrectly listed as “FXYB07.” The correct **Funding** statement appears below.

