# Supplementary material for: LC-MS/MS untargeted lipidomics uncovers placenta lipid signatures from intrahepatic cholestasis of pregnancy
Source: Front Physiol. 2024 Jun 3;15:1276722. doi: 10.3389/fphys.2024.1276722 (PMC11180999; doi:10.3389/fphys.2024.1276722)
Supplement: Supplementary file 4 [file DataSheet1.docx]

**
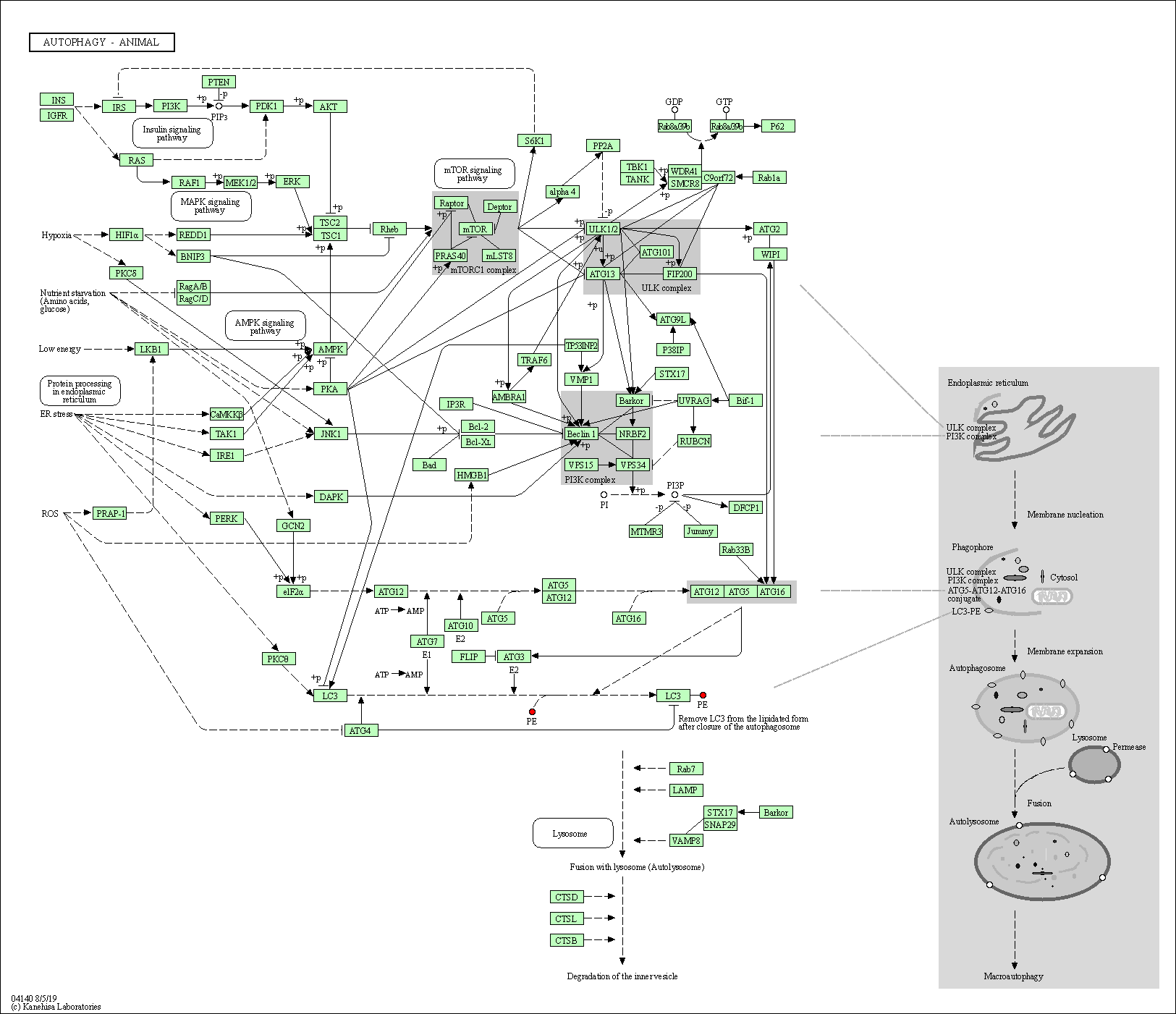
**

**Fig S1.** Analysis of differential PE lipids involved in autophagy pathway. PE, Phosphatidylethanolamine.


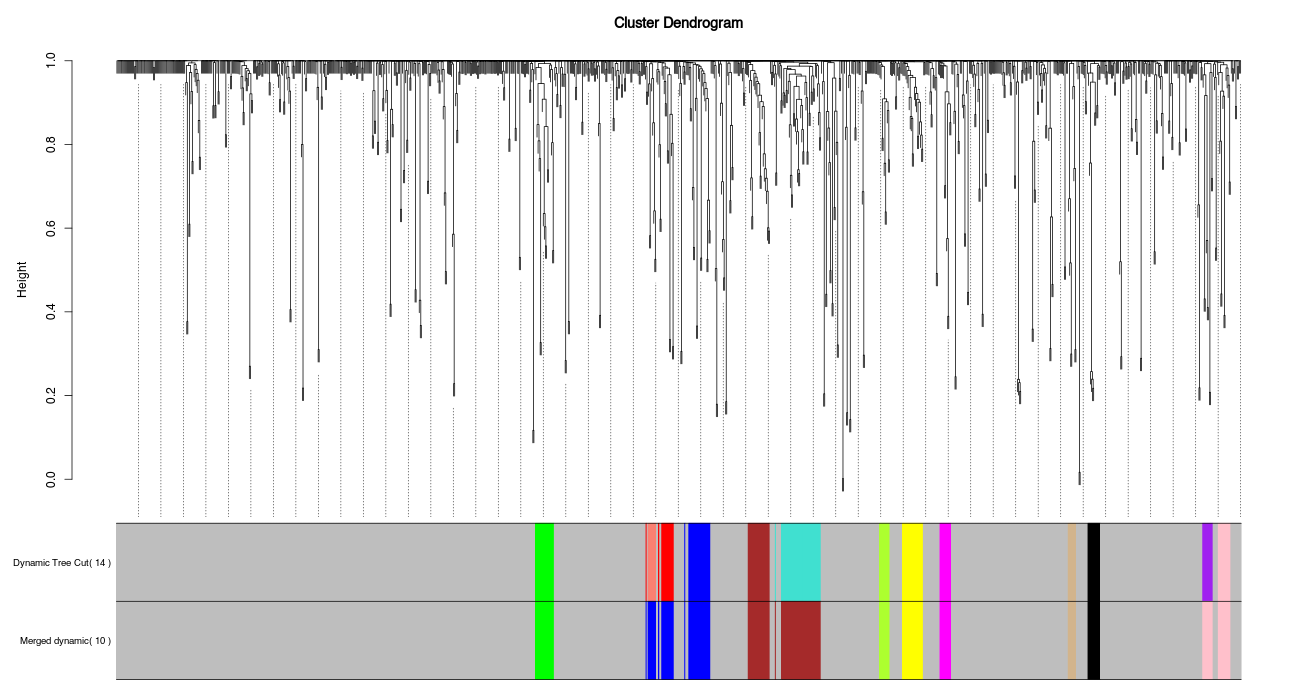


**Fig S2.** A module cluster tree was used to visualize the distribution of lipids in each module.


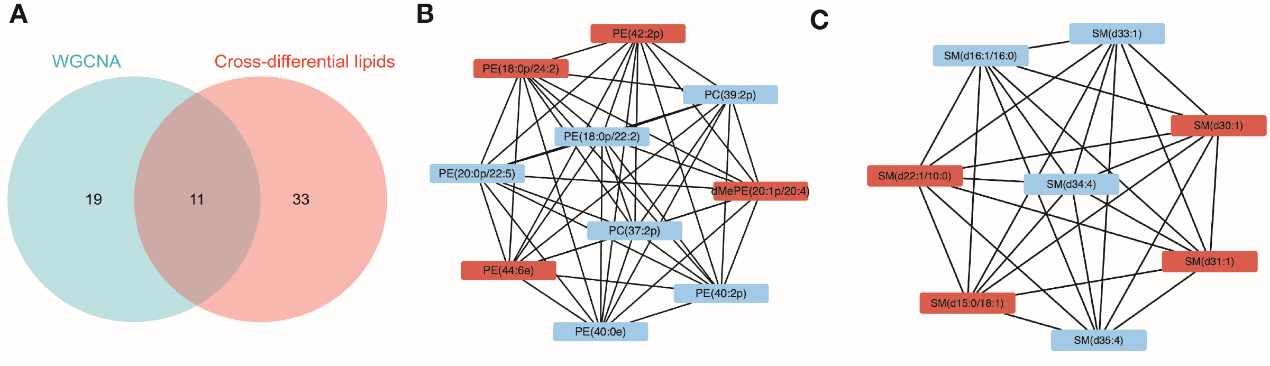


**Fig S3. (A)** Venn diagram showed there are 11 differentially co-expressed lipids in both mild and severe ICP group and the WGCNA analysis. Hub lipids in purple **(B)** and salmon **(C)** module was identified by CytoScape. WGCNA, Weighted correlation network analysis.


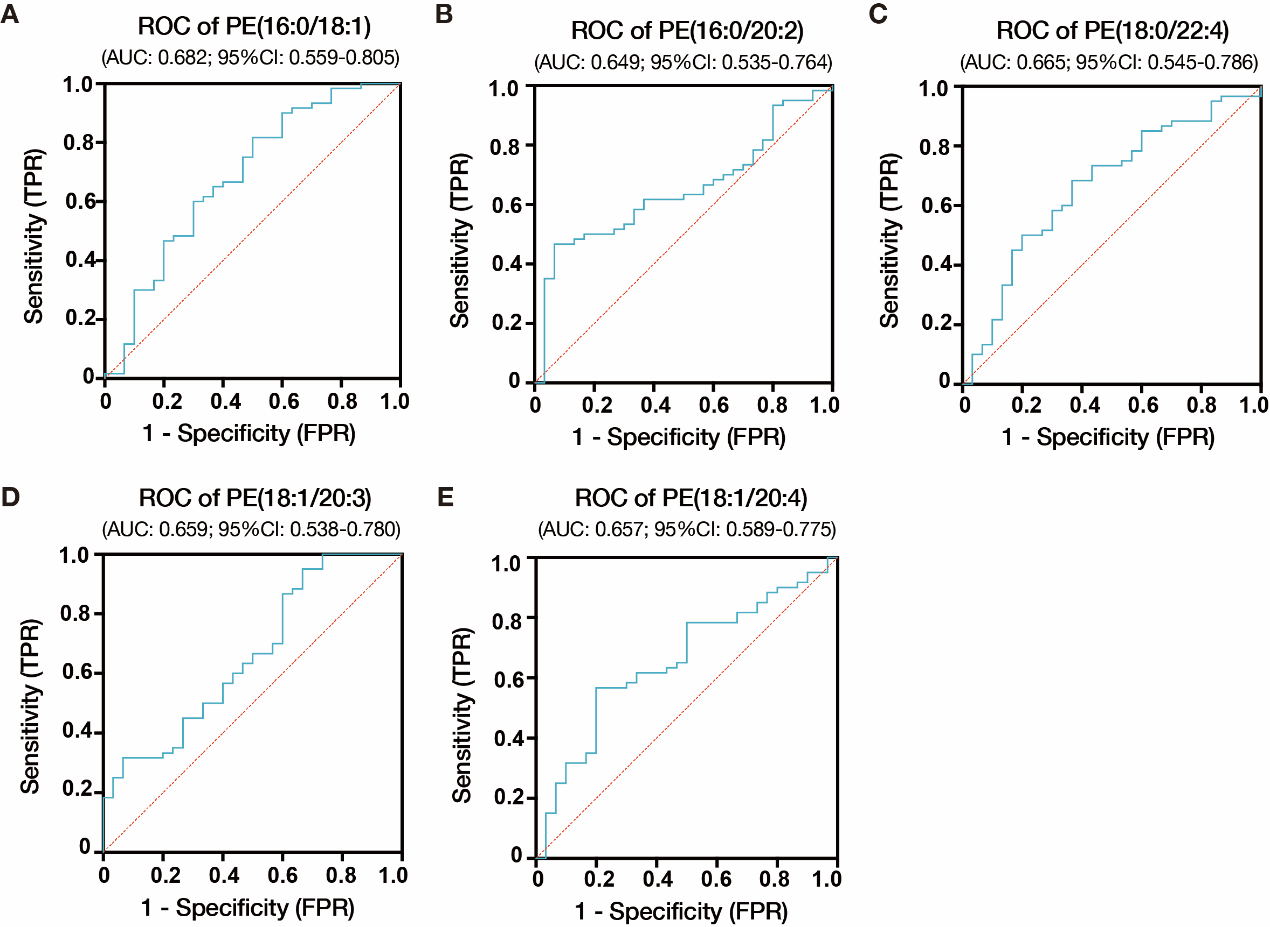


**Fig S4.** Diagnostic utility of 5 lipids identified from autophagy pathway. **(A)** ROC analysis of PE (16:0/18:1). **(B)** ROC analysis of PE (16:0/20:2). **(C)** ROC analysis of PE (18:0/22:4). **(D)** ROC analysis of PE (18:1/20:3). **(E)** ROC analysis of PE (18:1/20:4). AUC, area under the curve; CI, confidence interval; ROC, receiver operating characteristic.
